# The impact of early surgical ventricular septal defect repair on parenting behavior and mother-child relationship: a prospective longitudinal study

**DOI:** 10.3389/fped.2024.1455310

**Published:** 2024-10-23

**Authors:** Jonas Hemetsberger, Stefan Mestermann, Hannah Nicol, Ariawan Purbojo, Robert A. Cesnjevar, Oliver Kratz, Anna Eichler, Jennifer Gerlach

**Affiliations:** ^1^ Department of Child and Adolescent Mental Health, University Hospital Erlangen, Friedrich-Alexander-Universität Erlangen-Nürnberg (FAU), Erlangen, Germany; ^2^ Department of Pediatric Cardiac Surgery, University Hospital Erlangen, Friedrich-Alexander-Universität Erlangen-Nürnberg (FAU), Erlangen, Germany; ^3^ Department of Pediatric Cardiovascular Surgery, Pediatric Heart Center, University Children’s Hospital Zürich, Zürich, Switzerland

**Keywords:** congenital heart disease, ventricular septal defect, pediatric cardiac surgery, mother, mother-child relationship, parenting behavior, longitudinal study

## Abstract

**Introduction:**

Many studies have investigated the impact of congenital heart defects (CHD) on child development. However, because CHD not only affects the child and his or her development but, also the entire family, family functioning after pediatric cardiac surgery is of increasing research interest. This prospective childhood-adolescence case-control study aimed to examine differences and changes in parenting behavior and mother-child relationship quality after early surgical repair of an isolated ventricular septum defect (VSD) compared to non-affected controls.

**Patients and methods:**

39 affected children (*M* = 7.3 years) with surgically repaired VSD and their mothers were compared with a matched, non-affected control group of 39 mother-child-dyads (*M* = 7.3 years) during primary school age (t1). At child early adolescence, 24 affected children (*M* = 12.4 years) and 24 children of the control group (*M* = 13.2 years) were examined again (t2). Parenting behavior characteristics (t1: mother report; t2: mother- and child report) and mother-child relationship quality (t2: child report) were measured by standardized questionnaires.

**Results:**

The mother-rated parenting behavior dimensions Involvement (*p* < .001, *η^2^_p_* = .37), Parental Monitoring (*p* = .014, *η^2^_p_* = .17) and Corporal Punishment (*p* < .001, *η^2^_p_* = .57) significantly decreased from t1 to t2 in both cohorts. Responsible Parenting Behavior tended to decrease from t1 to t2 in the control group, while remaining stable in the VSD-group (*p* = .088, *η^2^_p_* = .09). Independent of the group, higher mother-child relationship quality was associated with more Positive Parenting Behavior (*p* < .001, *η^2^_p_* = .34), more Involvement (*p* = .003, *η^2^_p_* = .22) and fewer Inconsistency (*p* < .001, *η^2^_p_* = .31) in the child-rating; and more Positive Parenting Behavior in the mother-rating (*p* = .039, *η^2^_p_* = .10).

**Conclusion:**

VSD affected mother-child-dyads were mostly comparable in their parenting behavior characteristics and mother-child relationship quality to non-affected controls. The absence of a decrease in maternal Responsible Parenting Behavior in the VSD group may indicate challenges during the developmental task of autonomy in adolescence. Nevertheless, adaptive family functioning after early pediatric surgical VSD repair seems possible.

## Introduction

Occurring in one percent of newborns congenital heart defects (CHDs) are the most common form of organ mutation in humans (1), with approximately 90% of the affected children reaching adulthood (2). CHDs include various types of defects, ranging from mild to drastically severe (3). Of these, ventricular septal defects (VSDs) are the most common form of CHD, with a prevalence of 3 per 1,000 live births (4). Isolated VSDs are considered a “simple” form of severity compared to more complex forms such as tetralogy of Fallot or univentricular hearts (5). Due to medical advancements, morbidity and mortality rates as well as potential complications are considerably low and the morbidity and mortality rates have been drastically reduced in the last few decades. Thus, long-term physical outcomes after surgery are described as excellent (6). Although treated children with VSD are expected to have almost similar somatic developmental outcomes to non-affected children, little research has been devoted to the psychosocial adjustment of affected children. Recent studies have shown that psychosocial adjustment and a high quality of life after early VSD repair are possible, especially when mothers of affected children reported low anxiety levels and highly proactive and warm parenting styles (7, 8).

CHDs and the resulting need for early pediatric cardiac surgery can place a heavy burden on the whole family (7, 9) Parents of affected children may show signs of increased stress (increased cortisol concentrations) and anxiety levels (9–12), which may be explained by various factors such as receiving the initial diagnosis, the process of surgery, and separation from parents during treatment and lack of psychological support during this period (13). Families with fewer psychosocial resources and less social support are at an even greater risk of experiencing stress and lower quality of life in the following period (14). In addition, affected parents may develop long-term symptoms of post-traumatic stress disorder (PTSD) due to the diagnosis and associated stress (10). Clinical symptoms of trauma can be found in one-third of mothers and one-fifth of fathers one month after surgery (15). Next to increased stress levels, 18% of parents of children with CHD reported unusual levels of anxiety (16).

Impaired parental mental health may therefore influence the socioemotional, behavioral, and cognitive development of the child, for example through less sensitive parental care (17, 18). Ongoing trauma reactions can impair parents’ ability to be available and to provide sensitive and attentive care to their child and maternal anxiety is the most important risk factor for developing behavioral problems in children with CHD (19). Some studies have already demonstrated that parental stress and maternal anxiety increase the risk of behavior problems in CHD cohorts (19–21). In addition to surgery and subsequent medical treatment parental characteristics also have an additional influence on child development (22). Particularly when coping with early open heart surgery in early childhood, children rely on their parent's support in behavioral and emotional regulation (23), thus highlighting the role of parents for their affected children (7, 8, 24).

Based on the hypothesized underlying mechanism of parental characteristics influencing child development psychosocial stressors—such as parental psychopathology or poor health condition of the child—have a negative impact on mother-child interaction by impairing emotionally warm and sensitive interaction with their child, resulting in negative consequences for child development (10, 25, 26). Difficulties in mother-child interaction in families with CHD-affected children due to increased psychosocial stress have already been reported (26), and linked parental stress to altered parenting behaviors as well as dysfunctional infant brain development and behavioral, cognitive, and socio-emotional impairments in affected children (24, 27).

The existing literature provides mixed results on the association between the severity of CHD and the impact on parental outcomes. Some studies have demonstrated higher parental stress levels and more mental health problems with increasing severity of diagnosis (28–30), while in contrast, others reported no significant predictive value of CHD severity on parental outcomes (25, 31, 32).

Although VSD is considered to be a “simple” form of CHD with an almost similar somatic developmental outcome as described earlier (6), previous results examining our cohort of isolated VSD-affected children and their mothers demonstrated lower levels of maternal hair cortisol concentrations (hypocortisolism) during children`s primary school age (6–9 years) resulting in higher maternal stress levels (9). Furthermore, anxiety symptoms were elevated at primary school age when their mother had anxiety symptoms herself. However, positive parenting behaviors and a positive mother-child relationship could be protective factors and attenuate the impact of parental stress and anxiety on the child (7). This raises the question of whether parenting behaviors and the quality of the mother-child relationship are affected after the child's early surgical repair of VSD. As parenting behaviors and the mother-child relationship are important predictors for child development (17), this is crucial for later psychosocial adjustment.

Although other studies have investigated the impact of CHD on child development, little is known about characteristics of parenting behavior and parent-child relationship quality in primary school age and adolescence, with the focus of research predominantly being on infants and toddlers (33). Therefore, this prospective case-control study aimed to examine differences and changes in parenting behavior characteristics and mother-child relationship quality of the previously reported VSD-corrected group compared to a non-affected control group from primary school age to early adolescence (7–9).

## Methods

### Participants and study design

**Figure 1:**
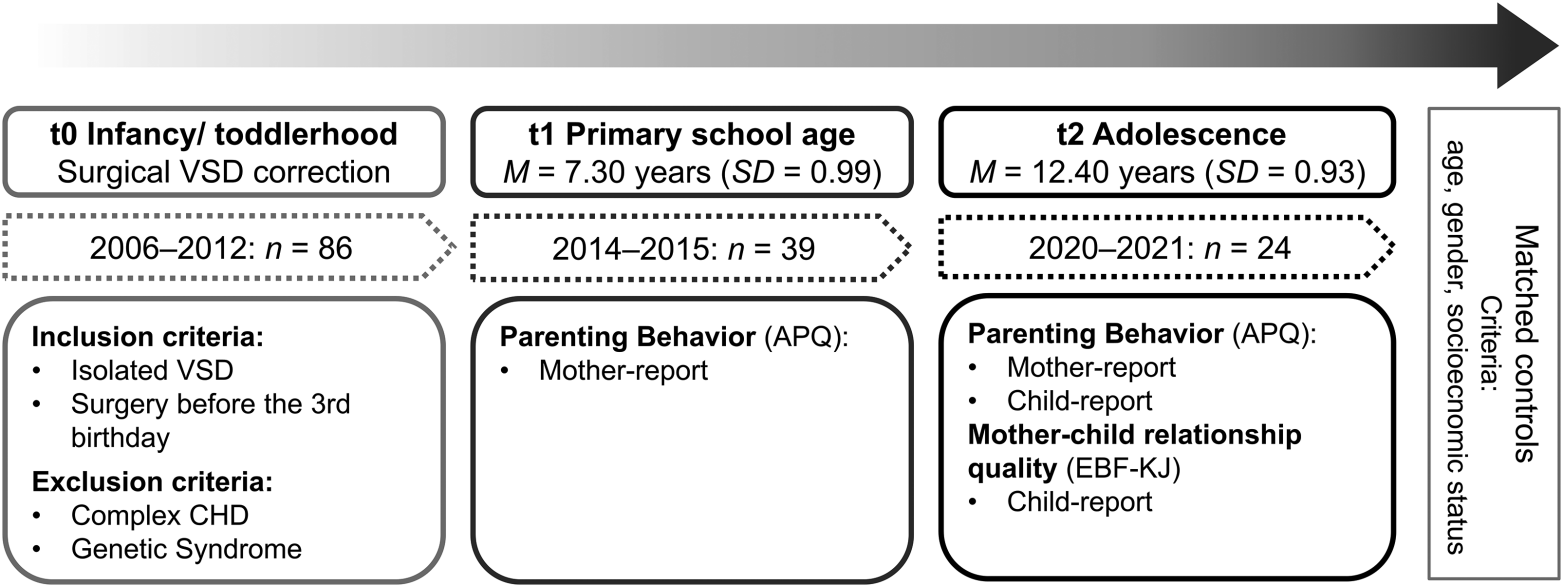
Study design. VSD, ventricular septal defect; CHD, congenital heart diseases; APQ, Alabama parenting questionnaire (34), EBF-KJ, Parent Image Questionnaire for Children and Adolescents (35).

The present study is part of a longitudinal research project of the Department of Child and Adolescent Mental Health and the Department of Pediatric Cardiac Surgery at the University Hospital Erlangen, Germany (7). Aim of the longitudinal research project was to investigate the long-term psychological consequences of early surgical correction of isolated VSD from child primary-school age to early adolescence. From March 2006 to March 2012, 86 children underwent surgery for isolated VSD before their third birthday (t0, see Figure 1).

In 2015, families were invited to participate in the follow-up study. Children with a genetic syndrome (e.g., Down syndrome; *n* = 14), additional congenital malformations (e.g., VACTERL association; *n* = 5), and complex heart defects (e.g., tetralogy of Fallot; *n* = 6) were excluded to provide a homogenous cohort without data corruption by complex differences of the underlying heart disease. Families whose current address could not be determined (*n* = 6) were also excluded from the sample. One child died from non-cardiological causes. The remaining 54 families were contacted by mail, of which 39 families participated in the first data collection at child primary-school age (t1, survey period 2014–2015; drop-out rate 28%). In 2020, the *n* = 39 families were contacted again and invited to a follow-up survey in early adolescence (t2). Of these, *n* = 24 children participated with their mothers in the second data collection (drop-out rate 38%). At both measurement times, the VSD group was matched with a non-affected control group for sex, age, and socioeconomic status recruited from the Franconian Cognition and Emotion Studies child and adolescent general-population sample (36). More detailed information on the study cohort the control group was recruited from can be found in the related study protocols (8, 9). Descriptive data and a comparison between the VSD-group and the control group are presented in Table 1. The adolescents in the VSD group were about 1 year younger than the control group at t2. Our data collection took place during the COVID-19 pandemic, which caused some delays in the course of the study, resulting in the age difference.

**Table 1:** Child and family descriptive data at t2 (adolescence) in VSD (*n* = 24) and control group (*n* = 24).

	VSD		Controls		Statistics			
	*M*/*n*	*SD*/%	*M*/*n*	*SD*/%	*t*/*χ*^2^	*df*	*p*	*d*/*φ*
Child								
Age	12.4	0.93	13.2	0.24	−4.08	46	<0.001***	0.68
Sex								
Male	11	45.8	10	41.7	0.09	1	0.771	0.04
Female	13	54.2	14	58.3				
Country of birth								
Germany	23	95.8	24	100	1.02	1	1.00	0.15
Other Country	1	4.2						
School level								
High	8	33.3	11	45.8	4.23	4	0.319	0.30
Medium	6	25	9	37.5				
Low	8	33.3	4	16.7				
Other	2	8.4	0	0				
Characteristics of VSD surgery								
Age at surgery (months)	12.78	10.38	–	–	–	–	–	–
Post-surgical hospital stay (nights)	7.87	3.22	–	–	–	–	–	–
Complications after VSD surgery^a^								
Cardiac arrythmia	3	13.04	–	–	–	–	–	–
AV block	1	4.40	–	–	–	–	–	–
Left bundle branch block	1	4.40	–	–	–	–	–	–
Inflammation of the scar	1	4.40	–	–	–	–	–	–
Fluid retention (need for punctuation)	1	4.40	–	–	–	–	–	–
Need for cardiac pacemaker	1	4.40	–	–	–	–	–	–
Family								
socioeconomic status^b^	11.08	2.55	11.63	2.50	0.74	46	0.460	0.21
Family size (number of people living in the household)	3.80	1.01	3.96	0.75	−0.60	42	0.553	0.18

School level: High = 13 years, medium = 10 years, low = 9 years of education.

^a^ Multiple answers possible.

^b^ Socioeconomic status: sum index: maternal/paternal education level [<9(1), 9(2), 10–12(3), or 13(4) years of education), maternal/paternal origin [international (0) or national (1)], monthly family income (<EUR 1,000 (1), EUR 1,000–2,000 (2), EUR 2,000–3,000 (3), EUR 3,000–4,000 (4), EUR 4,000–5,000 (5), >EUR 5,000 (6)], theoretical range 6–16.

*** *p* < 0.01.

At t1 and t2, mothers filled out questionnaires on their parenting behavior; at t2, adolescents answered questionnaires on the mother-child relationship quality and maternal parenting behavior.

The study protocol was approved by the local ethics committee of the Medical Faculty of the University of Erlangen-Nürnberg (t1: 4,596, 2 April 2014; t2: 353_18B, 12 April 2019), and was conducted in accordance with the Declaration of Helsinki. All mothers gave written informed consent for the study as well as for publication of the results, all children gave informed assent.

### Measures

#### Maternal parenting behavior

The German 40-item version of the Alabama Parenting Questionnaire (APQ) (34, 37) for parents (t1 and t2), and the 41-item version for their children (t2) is well-validated and was used to assess maternal parenting behavior dimensions in a pen-and-paper format. For the mother, the APQ captures the dimensions of Inconsistency (6 items), Positive Parenting Behavior (6 items), Involvement (6 items), Low Monitoring (6 items), Corporal Punishment (4 items), Powerful Enforcement (6 items), and Responsible Parenting behavior (6 items). For the child, the APQ captures the dimensions of Inconsistency (6 items), Positive Parenting Behavior (8 items), Involvement (17 items), and Low Monitoring (10 items) on four subscales. Each item is assessed using a five-point Likert scale [“almost never” (1) to “almost always” (5)], resulting in subscale mean value ranges from 1 to 5. The dimension of Inconsistency assesses parenting behavior that lacks consistency, such as threatening to punish a child but failing to follow through. Realibility for Inconsistency is high (Cronbach's *α* = 0.72). Positive Parental Behavior (*α* = 0.84) involves emotional warmth and child-centered interactions, such as praising a child for doing well. Involvement (*α* = 0.66) measures active parental support for a child's development through participation in activities, such as helping with homework. The dimension Low Monitoring (*α* = 0.75) is used to measure the extent to which parents lack information about their child's social contacts and activities, for instance, if a child is out of the house and the parent is unaware of their whereabouts. The dimension Corporal Punishment (*α* = 0.60) includes examples such as holding children down or shaking them when they do something wrong. Powerful Enforcement (*α* = 0.71) examines an authoritarian parenting style and is described as overreacting and emotionally negative. Responsible Parenting Behavior (*α* = 0.72) captures a conscious educational attitude of the parent, such as discussing free time activities with the child (37).

#### Mother-child relationship quality

The child-rated mother-child relationship quality was assessed using the Parent Image Questionnaire for Children and Adolescents (EBF-KJ) (35) in a pen-and-paper format. The questionnaire consists of three resource dimensions (Cohesion: 5 items, Identification: 4 items, Autonomy: 4 items) and five risk dimensions (Conflicts, Rejection/Indifference, Punishment, Emotional Appropriation, Fears/Overprotection). Each item is rated using a five-point Likert scale [“never” (1), “rarely” (2), “sometimes” (3), “often” (4), “always” (5)]. For each dimension, a mean total score was calculated and transformed into sex- and age-standardized T-Scores (*M* = 50, *SD* = 10). To calculate the score for the mother-child relationship quality, the sum of the five risk dimensions was subtracted from the sum of the three resource dimensions, resulting in an index value (*T*-values with a theoretical range between 0 and 84). *T*-Scores < 35 indicate strained, lower relationship quality, and high *T*-Scores > 65 indicate above-average positive relationship quality (38). The overall reliability of the EBF-KJ is high (*α* > 0.80).

#### Socioeconomic status

Socioeconomic Status was assessed by creating a sum index based on parental education level [four categories: <9 (1), 9 (2), 10–12 (3), or 13 (4) years of education], parental origin [two categories: international (0) or national (1)] and monthly family income [six categories: <EUR 1,000 (1), EUR 1,000–2,000 (2), EUR 2,000–3,000 (3), EUR 3,000–4,000 (4), EUR 4,000–5,000 (5), >EUR 5,000 (6)] with a theoretical range from 6 to 16 (7).

### Statistical analyses

Statistical analyses were conducted using IBM® SPSS® Statistics version 28 (IBM Corporation, Armonk, NY, USA, 2021). Normal distribution (Shapiro-Wilk test) and variance homogeneity (Levene's test) were tested without any abnormalities. Two-sided tests were performed, and *α* = 5% was specified (*p* < .05 = significant, *p* < .01 = highly significant, *p* < .10 = trend significant). Due to the small sample size, we also reported trend significance. Effect sizes (Phi Coefficient *φ*, Cohen's *d*, Partial Eta Squared *η^2^_p_*) were expressed in absolute numbers and interpreted as follows: *η^2^_p_* ≥ .01 small, *η^2^_p_* ≥ .06 medium, *η^2^_p_* ≥ .14 large effect; *d* ≥ .20 small, *d* ≥ .50 medium, *d* ≥ .80 large effect; *φ* ≥ .10 weak, *φ* ≥ .30 medium, and *φ* ≥ .50 strong association (39). For hypotheses testing, first, asking for parenting behavior differences between the VSD- and control group in primary-school age (t1) and adolescence (t2), analyses of variance with repeated measures (ANOVA_rm_) were conducted for each dimension of the mother-rated parenting behavior questionnaire (between-subject factor: VSD group yes/no; time factor: primary-school age t1, adolescence t2). The group main effect (is there a general difference between VSDs and controls in the parenting behavior dimension?), the time main effect (is there a general developmental effect in the parenting behavior dimension independent of group status?), and the group*time interaction (is there a developmental time-effect for only one group?) effect were interpreted. When interaction effects were significant, *post hoc* comparisons (*t-tests*) were calculated. Second, unpaired *t*-tests were calculated to compare VSD-affected children with non-affected controls in terms of maternal parenting behavior dimensions and mother-child relationship quality. Third, asking for effects of maternal parenting behavior dimensions on child-rated mother-child relationship quality, analyses of covariance (ANCOVA) were calculated with the mother-child relationship quality as a dependent measure (between-subject factor: VSD group yes/no; independent measures/covariates: mother-/child-rated parenting behavior at t1 and t2) with *post hoc* pair-wise *t*-tests were interaction effects were significant.

## Results

### Descriptive data

Sample characteristics are depicted in Table 1. Adolescents in the VSD-group were younger (*M* = 12.4, *SD* = 0.93) than in the non-affected control group (*M* = 13.2, *SD* = 0.24; *p* < .001). Descriptive parenting behavior data can be found in Table 2.

**Table 2:** Questionnaire descriptive data and mean differences in mother-rated maternal parenting behavior dimensions between groups (VSD vs. controls) and over time (t1 to t2), ANOVA_rm_ results.

Maternal parenting behavior	VSD		Controls		Statistics								
					Group			Time			Group × time		
Mother-rated	*M*	*SD*	*M*	*SD*	*F*	*p*	*η* ^*2*^_*p*_	*F*	*p*	*η* ^*2*^_*p*_	*F*	*p*	*η* ^*2*^_*p*_
Inconsistency_t1_	2.48	0.46	2.39	0.31	0.41	.525	0.01	0.01	0.944	0.00	0.01	0.944	0.00
Inconsistency_t2_	2.43	0.73	2.41	0.59									
Positive parenting behavior_t1_	4.43	0.42	4.31	0.24	1.19	0.283	0.04	0.09	0.765	0.00	0.09	0.765	0.00
Positive parenting behavior_t2_	4.47	0.48	4.28	0.36									
Involvement_t1_	4.21	0.54	4.15	0.39	0.01	0.932	0.00	18.47	0.001*	0.37	0.03	0.874	0.00
Involvement_t2_	3.78	0.65	3.81	0.43									
Low monitoring_t1_	1.17	0.20	1.20	0.29	2.28	0.141	0.07	6.70	0.014**	0.17	0.93	0.343	0.03
Low monitoring_t2_	1.33	0.27	1.43	0.37									
Powerful enforcement_t1_	3.09	0.46	2.87	0.32	0.03	0.858	0.00	0.07	0.787	0.00	3.73	0.062***	0.10
Powerful enforcement_t2_	2.88	0.63	3.02	0.49									
Responsible parenting behavior_t1_	3.51	0.51	3.47	0.38	2.16	0.151	0.06	5.81	0.022**	0.15	3.09	0.088***	0.09
Responsible parenting behavior_t2_	3.47	0.68	3.23	0.42									
Corporal punishment_t1_	1.64	0.39	1.70	0.40	0.01	0.913	0.00	44.53	0.001*	0.57	1.16	0.290	0.03
Corporal punishment_t2_	1.35	0.37	1.28	0.29									
Child-rated (t2)							*t*				*p*		*d*
Inconsistency	2.27	0.61	2.32	0.72			0.04				0.810		0.07
Positive parenting behavior	3.91	0.63	3.57	0.58			−1.59				0.073***		0.54
Involvement	3.40	0.65	3.17	0.48			−1.21				0.194		0.40
Low monitoring	1.87	0.57	1.71	0.54			−0.81				0.336		0.30
Mother-child relationship quality	55.96	12.15	50.88	9.22			−1.62				0.112		0.47

Maternal Parenting Behavior: APQ, Alabama Parenting Questionnaire (*n*_child_ = 42; *n*_mother_ = 42). Frick; Reichle & Franiek (34, 37), mean subscale sums (theoretical range: 1–5). Mother-child relationship quality: EBF-KJ = Parent Image Questionnaire for Children and Adolescents (*n* = 47) (35), *T*-Score (*M* = 50, *SD* = 10).

* *p* ≤ 0.01.

** *p* ≤ 0.05.

*** *p* ≤ 0.10.

### Developmental change (t1 to t2) and group differences (VSD vs. controls) in mother-rated maternal parenting behavior

**Figure 2:**
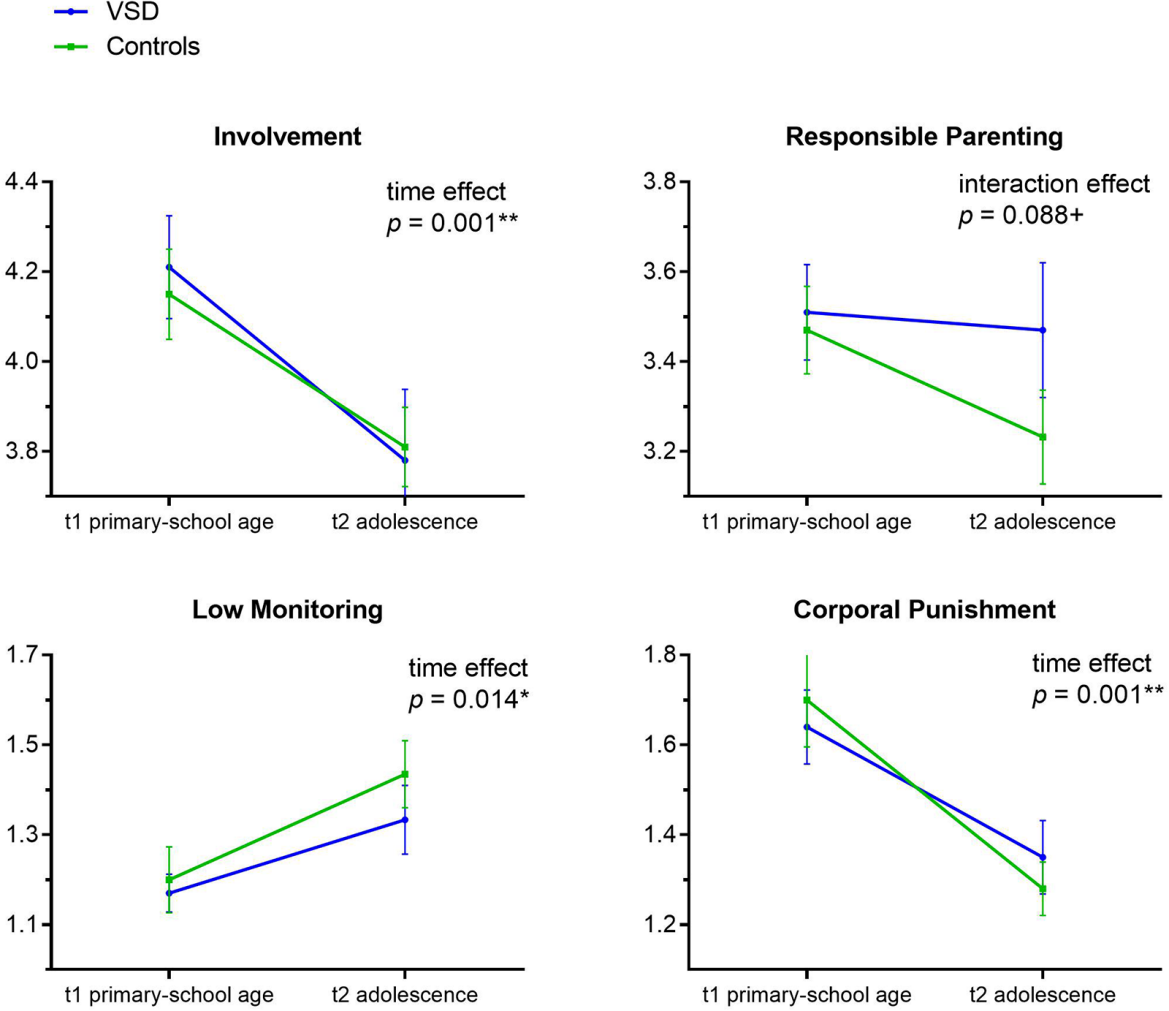
Developmental changes in maternal parenting behaviour dimensions from primary-school age (t1) to adolescence (t2) for significant ANOVA results. Alabama Parenting Questionnaire Subscale Scores, theoretical range 1–5 (37).

ANOVA_rm_ results are presented in Table 2. There were no significant time, group, or interaction effects for the parenting dimensions Inconsistency and Positive Parenting. Thus, there was no general developmental effect from primary school age to adolescence or a VSD vs. control group difference at no measurement time point. Significant general developmental effects—independent of VSD status—from t1 to t2 were found for Involvement (*p* < .001, *η^2^_p_* = .37), Responsible Parenting (*p* = .022, *η^2^_p_* = .15), Monitoring (*p* = .014, *η^2^_p_* = .17) and Corporal Punishment (*p* < .001, *η^2^_p_* = .57): These four parenting strategies decreased from t1 to t2 for all study participants (see Figure 2). The effects were large. For Powerful Enforcement and Responsible Parenting, there were interaction effects by trend (*p* = .062, *η^2^_p_* = .10/*p* = .088, *η^2^_p_* = .09) at medium effect size. There were no significant *post hoc* results for Powerful Enforcement. However, *post hoc* tests revealed that Responsible Parenting differed in adolescence between the VSD-group and controls (*p* = .051, *η^2^_p_* = .11) and the decrease was only significant for controls (*p* < .05, *η^2^_p_* = .20), whereas it remained stable over time in the VSD-group (see Figure 2).

#### *Post hoc* power analyses

*Post hoc* power analyses for ANOVA_rm_ and within-between subject factor interaction with a sample size of *n* = 48 and an *α* error probability of 0.05 revealed observed effect sizes in the range of 0.09 < *η^2^_p_* < 0.57 statistically relevant effects with a statistical power (1 – *β*) of > 0.98.

### Group differences (VSD vs. controls) of child-rated maternal parenting behavior in adolescence

Unpaired *t*-tests revealed that adolescents of the VSD-group reported their mothers showed more Positive Parenting Behavior than adolescents in the control group by trend with medium effect size [*t*(48) = −.159, *p* = .073, *d* = 0.54; Table 2]. There were no other significant differences in child-rated maternal parenting behaviors showing that both groups rated their mothers as comparable in behaviors such as Inconsistency, Involvement, and Low Monitoring.

#### *Post hoc* power analyses

*Post hoc* power analysis for unpaired *t*-tests with a sample size of *n* = 48 and an *α* error probability of 0.05 revealed the observed effect size *d* = 0.54 statistically relevant effects with a statistical power (1 – *β*) of 0.45.

### Group differences in child-rated mother-child relationship quality under consideration of parenting behavior

Table 3 presents the pre-correlations of mother-child relationship quality dimensions [child-rating, (t2)] and parenting behavior dimensions [mother-ratings (t1, t2) and child-rating (t2)]. The following parenting behavior dimensions were added as covariates to the ANCOVA analysis: Mother-rating (t2): Positive Parenting; child-rating (t2): Inconsistency, Positive Parenting and Involvement.

**Table 3:** Pearson correlations of mother-child relationship quality (total score, t2) with maternal parenting behavior dimensions.

Maternal parenting behavior	t1 Primary-school age	t2 Adolescence	
	Mother-rating	Mother-rating	Child-rating
Inconsistency	−0.32	−0.18	−0.53*
Positive parenting behavior	0.17	0.37**	0.59*
Involvement	0.16	0.01	0.50*
Low monitoring	−0.05	−0.06	−0.24
Corporal punishment	−0.11	−0.20	
Powerful enforcement	0.16	0.11	
Responsible parenting behavior	0.02	0.04	

Maternal Parenting Behavior: APQ, Alabama Parenting Questionnaire (34, 37). Mother-child relationship quality: EBF-KJ, Parent Image Questionnaire for Children and Adolescents (35). Sample size: t1 *n* = 37, t2 *n* = 43–45.

* *p* ≤ 0.01.

** *p* ≤ 0.05.

Table 4 shows the ANCOVA results, analyzing the effects of maternal parenting behavior dimensions on child-rated mother-child relationship quality in separate analyses. Independent of group, less Inconsistency [*F* (1, 40) = 17.92, *p* < .001, *η^2^_p_* = .31], more Positive Parenting [*F* (1, 41) = 20.83, *p* < .001, *η^2^_p_* = .34] and more Involvement [*F* (1, 38) = 10.40, *p* < .05, *η^2^_p_* = .22] in the child-rating were associated with higher mother-child relationship quality; the results were significant and of large effect size. At medium effect size, the maternal Positive Parenting rating was significantly positively associated with mother-child relationship quality [*F* (1, 39) = 4.54, *p* < .05, *η^2^_p_* = .10]. Meaning, independent of VSD-status, for all study participants named parenting behavior dimensions correlated with a positive mother-child relationship in adolescence. Furthermore, analyses revealed a marginal group effect, when child-rated Inconsistency was added as a covariate: The mother-child relationship quality rating was higher for VSD-affected adolescents than for controls [*F* (1, 40) = 2.87, *p* = .098, *η^2^_p_* = .07; see Table 4]. The effect was of medium size. There were no significant covariate × group interaction effects for any of the parenting behavior dimensions, including Low Monitoring, Inconsistency, Corporal Punishment, Powerful Enforcement, and Responsible Parenting Behavior (*p* = .184–.822). This indicates that the effects of parenting behavior on the mother-child relationship were independent of group status.

**Table 4:** Mean group differences (VSD vs. controls) in child-rated mother-child relationship quality at t2 (adolescence, total score) under consideration of parenting behavior dimensions at t2 (covariates) in separate ANCOVAs (*F, p, η^2^_p_*).

	Covariate			Group			Covariate × group		
Child-rated parenting behavior									
Inconsistency	17.92	0.001*	0.31	2.87	0.098***	0.07	1.83	0.184	0.04
Positive parenting behavior	20.83	0.001*	0.34	0.76	0.389	0.02	1.83	0.184	0.04
Involvement	10.40	0.050**	0.22	0.01	0.906	0.00	0.51	0.822	0.00
Mother-rated parenting behavior									
Positive parenting behavior	4.54	0.050**	0.10	0.14	0.711	0.00	0.00	0.628	0.00

Maternal Parenting Behavior: APQ, Alabama Parenting Questionnaire (34, 37). Mother-child relationship quality: EBF-KJ, Parent Image Questionnaire for Children and Adolescents (35). Sample size: *n* = 23–24 controls vs. *n* = 19–21 VSD.

* *p* ≤ 0.01.

** *p* ≤ 0.05*.*

*** *p* ≤ 0.10.

#### *Post hoc* power analyses

*Post hoc* power analyses for ANCOVAs with a sample size of *n* = 45 and an *α* error probability of 0.05 revealed observed effect sizes in the range of 0.07 < *η^2^_p_* < 0.31 statistically relevant effects with a statistical power (1 – *β*) of 0.44–0.99.

## Discussion

Our study aimed to determine potential differences in parenting behaviors and the mother-child-relationship between mother-child-dyads with VSD-affected children in comparison to a non-affected control group.

### Stability and change of maternal parenting behavior in children with early surgical VSD repair and controls

#### Inconsistency

Our results showed that, in both groups, mother-rated Inconsistency remained stable from t1 to t2. Moreover, maternal inconsistency scores in our sample varied on a medium level, indicating a stable level of inconsistent discipline strategies in both groups from primary school to adolescence. Other studies investigating the effects of inconsistent parenting behavior on child development also found that inconsistent parental behavior towards children remained stable over time (40, 41). Research on personality traits may provide a possible explanation for the stability of inconsistent parental behavior over time: According to this, inconsistent behavior is associated with emotion dysregulation, especially in parent-child interactions, as found in individuals with certain personality traits, such as a more neurotic personality (42, 43). Personality traits are thought to be stable, especially in adulthood (44). Thus, certain personality traits may be related to more inconsistent parental behavior and may explain the stability of this behavior and should be included in future studies.

#### Positive parenting behavior

Furthermore, maternal Positive Parenting Behavior remained stable from child primary-school age through adolescence in both groups. Regardless of group, all mothers reported themselves as highly emotionally warm and supportive in interaction with their children. Also, previous research has reported on the stability of positive, sensitive, and supportive parenting behaviors (45–47). Our data suggest that a high level of Positive Parenting Behavior appears to be relevant throughout the developmental period from primary school age to (early) adolescence. Other studies also emphasize the important role of parental warmth and sensitivity for child development, both in normal and at-risk samples such as children with surgically repaired VSDs (7–9, 17, 18).

#### Involvement and monitoring

Both, maternal Involvement and Monitoring decreased (or rather the values in Low Monitoring increased) from child primary-school age to adolescence. A possible explanation is that both parenting behavior dimensions are based on child's age as well as developmental status and tasks. Whereas Involvement reflects parenting behavior of active support in child development and is represented in participating in child activities, Monitoring describes the parent's knowledge and information about the child's activities and social contacts. In child adolescence, mothers might gradually reduce their Involvement and Monitoring as adolescents become increasingly independent and strive for more autonomy. In addition, children in primary school age allow their parents to be more involved in their own lives than they do when children become adolescents (48). A decrease in parental Involvement and Monitoring with increasing age appears to be important for adolescents’ development (49, 50). The developmental task of autonomy in adolescence is underlined once again by findings that show that continued or even increased Involvement during adolescence has a counterproductive effect on adolescent well-being (51). Our findings of a decrease in maternal Involvement and Monitoring in both groups show that the detachment process in adolescents who underwent surgical VSD repair is age-appropriate and develops normally compared to non-affected controls.

#### Corporal punishment

Our results also showed a significant decrease in Corporal Punishment from child primary school age to adolescence from the mothers’ perspective. Overall and in both groups, mothers reported very low levels of Corporal Punishment at t1 and t2, meaning that mothers in this sample reported rarely using behaviors such as shaking, hitting, or grabbing to discipline their child; and that low use of Corporal Punishment decreased to an even lower level from t1 to t2. Looking at the results in a developmental context, age could be an explanation for the decrease in Corporal Punishment. Adolescents have grown in physical strength and size compared to children in primary school age, so mothers may find less Corporal Punishment appropriate during discussions. Another possible explanation could be that rates of Corporal Punishment are higher in (early) childhood than in adolescence (52) because parents might feel it is a more appropriate discipline strategy at younger child age than later in adolescence (53).

#### Powerful enforcement

Powerful Enforcement reflects parenting behavior of a more authoritarian parenting style (49) as it includes overreactive behavior and strict implementation of family rules in combination with an emotionally negative atmosphere in the family. For this parenting behavior dimension, we found a significant interaction between group and time. Our results suggest that Powerful Enforcement differs over time from the perspective of the mothers of children who underwent early pediatric cardiac surgery compared to the mothers of the non-affected control group. However, the pairwise comparisons of t1 and t2 in the VSD and control group were not significant. A possible explanation for this result could be the slightly modified test variables used in the ANOVA and *post hoc* analysis. The ANOVA tests whether the interaction between groups and Powerful Enforcement is significantly different from zero over time, while the *post hoc* analyses test whether the differences between the factors are significantly different from zero. Because the null hypotheses of the two analyses are different, the interaction and *post hoc* analyses may yield different results. One explanation for the non-significant pairwise comparisons could be the different sample sizes of the respective groups. Thus, we interpreted maternal Powerful Enforcement as rather comparable in both groups.

#### Responsible parenting behavior

Parents with high scores on Responsible Parenting Behavior describe themselves as highly proactive in child rearing, have a conscious educational attitude, and discuss free time activities with their children. In group comparisons, we found maternal Responsible Parenting Behavior to decrease over time only in the non-affected control group. In contrast, it was stable in the VSD group from child primary school age to adolescence. At least two possible explanations seem plausible. On the one hand, the age difference of almost one year between the two groups could explain the difference. Since children in the control group were already about one year older than children in the VSD group, the step towards emotional detachment from the family and home may have already taken place, and the parents may already be giving the adolescents more autonomy. On the other hand, mothers of children who underwent early pediatric cardiac surgery might want to be closer to their children based on potential remaining concerns for the child's physical health. In line with this thought, studies have shown higher levels of overprotectiveness and vigilance among mothers of children who underwent early pediatric cardiac surgery (54, 55). In contrast, mothers with typically developing children might go through the natural detachment process in adolescence which is also reflected in our data by a decrease in Responsive Parenting Behavior only in the control group.

In summary, our results indicate that mostly, mothers of children who underwent early pediatric cardiac surgery show comparable and as much adequate parenting behavior as mothers of non-affected controls. The only significant difference between the groups was in Responsible Parenting Behaviors during child adolescence. Therefore, the question remains as to why no group differences could be found in the present study regarding the remaining parenting behaviors recorded. Maternal adaptation in the group of heart-operated children could be a possible explanation: Despite difficulties in early childhood education and interaction (16, 33), mothers of heart-operated children seem to be able to educate their children during the primary-school years into early adolescence in the same way as mothers in the non-affected control group. This finding is consistent with previous studies reporting no difference in parenting between mothers with heart-operated children and non-affected children (54), as well as an improvement in the quality of life of parents of children with congenital heart defects with increasing time (56). It is possible that improved quality of life could be an indicator of parents’ development of adaptive capacity about their child's health status. In contrast, Involvement, Low Monitoring, and Corporal Punishment decreased from primary school age to early adolescence in both groups. The results of changing parenting behaviors are consistent with developmental psychological findings that assume higher aspirational autonomy in adolescents as they age (51). An adjustment in parenting behaviors may already occur in early adolescence and the pursuit of autonomy may also be of great importance in early adolescence. Inconsistency and Positive Parenting Behavior were found to be stable parenting practices. This could be attributed to parenting attitudes and personality structures (45–47).

In addition, only Responsible Parenting Behaviors in adolescents were found to differ between VSD and non-affected groups, whereas the other parenting behavior dimensions did not differ between groups. Overall, the existing literature showed less engagement of parents of children with cardiac surgery in early childhood (33, 47, 57); these studies focused on different congenital heart defects and not exclusively on isolated VSD. Another study found increased parent burden and reduced maternal engagement in interactions with their children, particularly after the time of diagnosis of the congenital heart defect and the child's cardiac surgery (15). In previously published data of the present study at child primary school age, mothers of children who underwent early pediatric cardiac surgery showed an increased physiological stress response (12), suggesting that mothers of children who have undergone early pediatric cardiac surgery are particularly vulnerable (9). Additionally, parental stress is positively associated with the severity of the child's heart defect (58). In turn, high levels of psychosocial stress may have a negative impact on parenting behavior (17). However, at child primary school age and in adolescence, the present study could hardly find differences in parenting behaviors in mothers of children with a surgically repaired isolated VSD compared to non-affected controls, so the risk factor of cardiac surgery due to an isolated VSD does not seem to play a role in parenting behaviors, for at least several years after the pediatric cardiac surgery. In line with this thought, previous findings of our sample showed that affected mothers were well adapted regarding psychological and neurobiological stress levels, especially during adolescence, and thus probably had sufficient resources to engage in appropriate parenting behaviors (9). Even after parental stress experiences around the child's pediatric cardiac surgery in early childhood have occurred, there could be an adjustment in parenting behavior accompanied by an improvement in parental quality of life (56). Nevertheless, our results are not transferable to other or even more severe CHD, so that support for affected families through appropriate interventions may be necessary. Especially in the period before and after the child's heart surgery psychological support for parents might be crucial, in order to promote positive parenting behaviors and a secure caregiver-child attachments to enable a favourable developmental outcome for the affected child.

### Mother-child relationship in the VSD-group compared to non-affected controls and influence of maternal parenting behavior

#### Influence of parenting behavior from a mother's perspective

In both groups, a high level of mother-reported Positive Parenting Behavior at t2 was associated with a more positive mother-child relationship quality. The finding suggests that, in general, mothers can contribute to a better child-reported relationship quality through parenting behaviors like emotional warmth as well as friendly, child-centered interactions. In line with this, adolescent-reported maternal Positive Parenting Behavior was also associated with higher mother-child relationship quality. Our results are consistent with previous research reporting on positive relations between Positive Parenting Behavior and mother-child relationship (55).

Overall, from the mother's perspective, only Positive Parenting Behavior and no other parenting behavior dimensions were related to child-rated mother-child relationship quality. This could be due to the discrepancy between self-report and third-party reports regarding parenting behavior dimensions. Discrepancies between self-reports and third-party reports - also about parenting behavior and relationship quality - are also reported in other studies [e.g., (59)]. Another explanation could be that self-report questionnaires are not effective in capturing one's behavior because of biases such as social desirability (37), again fostering discrepancies between self- and third-party reports.

Currently, there are limited studies on parenting behavior and the mother-child relationship in families with children who underwent early pediatric cardiac surgery. Most of these studies are limited to early childhood (60, 61). This study aims to contribute to the still unclear research field of the unique relationship between parents and children with cardiac surgery in later childhood and early adolescence. However, there are still some unclear aspects regarding parenting behavior and the mother-child relationship in children and adolescents who underwent early pediatric cardiac surgery. Therefore, further research is necessary.

Additionally, the presented results have practical implications for support services aimed at parents and children who underwent early pediatric cardiac surgery. Previous studies have demonstrated the significance of information and support services provided by professionals for affected families, particularly during early childhood, to promote the development of a healthy parent-child relationship and positive parenting behavior (33). Furthermore, the available results suggest that mothers of children who underwent early pediatric cardiac surgery may have difficulties granting them complete autonomy, especially in late adolescence. Therefore, counseling services for parents of affected children could be beneficial.

#### Influence of adolescent-reported parenting behavior

In summary, the results indicate a close relation between adolescents’ perceptions of parenting behavior and mother-child relationship quality. From the adolescents’ perspective, Positive Parenting Behavior, Parental Involvement, and low Inconsistency in parenting behavior were needed for high mother-child relationship quality. This appears to be true for all adolescents, as the self-reported relationship quality of the heart-operated adolescents was comparable to that of the non-affected control group. Adolescents of the VSD-group even reported their mothers show more Positive Parenting Behavior, which might be explained by potential remaining concerns for the child's physical health as described earlier.

In contrast, studies focusing on the mother-child relationship in CHD-affected samples in early childhood reported a less secure attachment and unstable relationship between parent and heart-operated child (33, 57). In addition, previous research found increased stress levels and anxiety in parents of heart-operated children in early childhood (21). It is possible that authoritative parenting behavior – often reflected in high parental sensitivity - represents a protective factor against the influence of risks on child development (7, 17, 18) and favors the development of a positive mother-child relationship in adolescence. Moreover, high mother-child relationship quality in the EBF-KJ is characterized by dimensions such as emotional attachment, identification with parents, autonomy, and low levels of negative parenting behaviors such as punishment or rejection, so the association between relationship quality and Positive Parenting Behaviors seems plausible in terms of attachment theory and parenting behavior research (62).

Overall, our findings suggest that adolescents who underwent early cardiac surgery had a relationship quality with their mothers that was as good as that of non-affected adolescents. The descriptive data even showed slightly higher mother-child relationship quality ratings in the VSD-group compared to controls, but this difference did not reach statistical significance. A possible explanation could be the age difference between the VSD and control group at t2: The non-affected adolescents were about one year older than adolescents in the VSD-group, and descriptively reported more conflicts with their parents. One reason for this may be the more advanced stage of puberty compared to the one-year younger adolescents who underwent early pediatric cardiac surgery.

### Strengths and limitations

When examining parenting behavior, using both the mother's and child's perspectives provided an advantage in mitigating potential biases. However, the limited sample size restricts the generalizability of the findings. Although the sample size was small, it was possible to form a homogeneous group of children with early surgically corrected VSD and to compare their development to a non-affected, matched control group. Nonetheless, it was difficult to statistically detect actual differences between this group and the control group. In addition, the data collected are based solely on questionnaire data and thus only on the subjective views of the children and their mothers, taking into account social desirability bias. However, questionnaires are a very economical way of recording parenting behavior and relationship quality. In future studies, it would be valuable to include multi-informative approaches to examine differences and changes in parenting behavior and mother-child relationship quality. This could involve gathering information from other individuals close to the child, such as teachers or relatives. In addition, utilizing more objective measurement methods, such as behavioral observations and standardized interviews or interactional tasks would be beneficial.

Moreover, the present study did not examine the father's perspective. However, few studies found differences in maternal and paternal stress responses facing a child with chronic disease (63, 64). For this reason, and from a family systemic perspective, future studies should include fathers in their analyses to uncover potential differences in parenting of children undergoing early pediatric cardiac surgery.

Another issue to discuss is the small sample size. Consequently, the results and statistical power were limited. This was accepted in favor of the benefits and the intention of the project. With this in mind, the results should be interpreted with caution and larger sample sizes should be aimed for in the future.

### Conclusion

Mothers of children with early surgically corrected VSD showed comparable parenting behaviors to mothers of non-affected children. A favorable influence of Positive Parenting Behavior on the mother-child relationship can be assumed. In addition, from the affected adolescent's perspective, the quality of the relationship between mother and child seems to develop comparably to non-affected adolescents. The absence of a decrease in maternal Responsible Parenting Behavior in the VSD group may indicate challenges during the developmental task of autonomy in adolescence. Nevertheless, our findings suggest that an adaptive family functioning after early pediatric surgical VSD repair is possible.

## Data Availability

The raw data supporting the conclusions of this article will be made available upon reasonable request by the corresponding author. The data are not publicly available due to privacy restrictions and the data protection law in Germany.
